# Editorial: Horizon in frontotemporal lobar degeneration related disorder

**DOI:** 10.3389/fneur.2023.1127073

**Published:** 2023-02-07

**Authors:** Qianqian He, Min Chu, Liyong Wu

**Affiliations:** ^1^Affiliated Beijing Friendship Hospital, Capital Medical University, Beijing, China; ^2^Department of Neurology, Xuanwu Hospital, Capital Medical University, Beijing, China

**Keywords:** frontotemporal lobar degeneration (FTLD), amyotrophic lateral sclerosis (ALS), heterogeneity, neuroimaging, molecular imaging, genetics

Frontotemporal lobar degeneration (FTLD) is a group of clinically, genetically, and neuropathologically heterogeneous neurodegenerative syndromes characterized by progressive atrophy of the frontal and temporal lobes (1). It can lead to progressive cognitive impairment, with the main clinical characteristics including behavioral abnormalities, personality changes, and impaired social cognitive function, as well as language, executive, and motor dysfunction. In addition to clinical presentation, diagnostic methods emphasize the strong support provided by genetic, neuropsychological, and neuro/molecular imaging tools.

In this Research Topic, we aim to collect and present a series of articles investigating sensitive diagnostic markers associated with the disease and identify its potential pathogenesis. The five articles closely related to the topic emphasized the clinical evidence supporting the diagnosis of FTLD, including data from neuroimaging studies and recent advances in genetics.

Accurate and early diagnosis of patients with FTLD is challenging due to clinical and neuropsychological heterogeneity, as well as atypical and overlapping clinical manifestations. Non-invasive quantitative molecular imaging can assist in better selective visualization of molecular targets *in vivo* for characterizing pathological protein deposition, functional changes, and neuroinflammation in FTLD to support diagnosis. Wang R. et al. discussed advances in molecular imaging in familial FTLD and focused on its implication and prospects in differentiating specific mutations in GRN, MAPT, and C9orf72. Tau-PET using 18F-flortaucipir and 11C-PBB3 demonstrated the elevated tau position in patients with FTLD, which can help differentiate MAPT from GRN or C9orf72 in familial FTLD. In addition, dopamine transporter imaging using 11C-DOPA and 11C-CFT in PET and 123I-FP-CIT in SPECT revealed the disturbance of dopaminergic neurons in both the asymptomatic and symptomatic stages of familial FTLD. PET imaging using 11C-MP4A demonstrated reduced acetylcholinesterase (AChE) activity in patients with FTLD, while PET with 11C-DAA1106 and 11C-PK11195 revealed an increased level of microglial activation associated with neuroinflammation.

Mapping the brain connectome will help to make an early diagnosis and to predict the severity of behavioral variant FTD (bvFTD). Nigro et al. reviewed the related literature on graph theory based on neuroimaging data. The characteristics of brain network organization in bvFTD, semantic variant PPA (svPPA), and non-fluent/agrammatic variant PPA (nfvPPA) were summarized, and the specific global and local brain network changes in patients with FTLD were analyzed. Patients with bvFTD showed a lower mean clustering coefficient, global efficiency, and a higher characteristic path length (2). At the same time, the loss of hubs in different brain regions of patients with bvFTD, including the frontal gyrus (right superior frontal, inferior orbitofrontal gyri, left anterior cingulate cortex, and cuneus), basal ganglia, and limbic system, has been reported, with new hubs appearing in the orbital frontal and parietal-temporal brain regions. The graphic analysis indicators reflect the neuropsychological characteristics of patients with bvFTD and are associated with clinical symptoms such as cognitive, behavioral, social cognitive and executive impairment, attention disorder, apathy, and inhibition. Due to the limited amount of research, no definite conclusions related to svPPA and nfvPPA have been drawn in this study.

With the concept of ALS-FTD spectrum disorder presented at the International Research Symposium on FTD and ALS held in London, Canada in June 2015 (3), considerable progress has been made in understanding the intersection and overlap between FTD and ALS. In addition to the C9orf72 mutation that has been identified as a key shared pathogenic gene for ALS-FTD, TARDBP, CHCHD10, FUS, TBK1, SQSTM1, UBQLN2, and VCP, mutations have also been associated with ALS-FTD (4). Clinically, ALS and FTD are frequently found in the same family. The study by Tábuas-Pereira et al. included 57 carriers of the pathogenic mutation without GRN, MAPT, or C9orf72 pathogenic variants. Rare mutations, including ERBB4, ANG, CHRNA4, CHRNB4, SETX, and GLT8D1, were found and predicted to be pathogenic in genes previously associated only with ALS, providing additional evidence that the ALS gene may also be involved in the pathogenesis of FTD.

Based on a cohort of Chinese ALS patients, Feng et al. completed pathogenic gene screening using second-generation sequencing technology, reported the first VCP mutation carrier of PDB showing ALS in Chinese population, and detailed the clinical characteristics of 3 ALS patients carrying the VCP p.R155C mutation. In this review, ALS patients with VCP p.R155C mutations tended to develop at a relatively young age, presenting with symmetrical proximal muscle weakness in the arms or legs, and then progressing to the distal limb muscles. ANXA11 pathogenic genes were investigated in the Chinese ALS and/or FTD cohort by Wang Y. et al. and the authors found a heterozygous mutation of ANXA11 (c.119A>G, p.D40G). This type of mutation was previously thought to be only associated with ALS and was first identified in ALS-FTD (5). A literature review found that patients with the same D40G mutation had different clinical symptoms. The clinical heterogeneity of ANXA11 mutation-related diseases is high, and further research is needed to advance this area.

In summary, FTLD is a heterogeneous disorder with a wide range of clinical, genetic, and neuropathological features. Our Research Topic presents the latest evidence from recent genetic and neuroimaging findings, focusing on the validation of proposed biomarkers from new perspectives and the discovery of new candidates. In the absence of clear, sensitive, and early biomarkers, it is suggested that a framework of diagnosis be established based on effective clinical criteria and practical and readily available diagnostic methods.

## Author contributions

All authors listed have made a substantial, direct, and intellectual contribution to the work and approved it for publication.
